# Study on the Nonlinear Conductivity of SiC/ZnO/Epoxy Resin Micro- and Nanocomposite Materials

**DOI:** 10.3390/ma12050761

**Published:** 2019-03-05

**Authors:** Haitao Hu, Xiaohong Zhang, Dingping Zhang, Junguo Gao, Chunxiu Hu, Yayun Wang

**Affiliations:** 1 Key Laboratory of Engineering Dielectrics and Its Application, Ministry of Education, Harbin University of Science and Technology, Harbin 150080, China; huhaitaodianqi@hrbust.edu.cn (H.H.); t_paris@163.com (D.Z.); gaojunguo@hrbust.edu.cn (J.G.); huchunxiu@hec-china.com (C.H.); 18846043117@163.com (Y.W.); 2 Harbin Electric Machinery Company Limited, Harbin 150040, China

**Keywords:** composite materials, silicon carbide, nano-ZnO, micro-ZnO, conductivity characteristics, nonlinear coefficient

## Abstract

To investigate the inhomogeneous distribution of electric fields in insulating equipment and components, five nonlinear-conductance composite materials based on epoxy resin (EP) (nano-SiC/EP, nano-ZnO/EP, micro-ZnO/EP, nano-SiC/ZnO/EP, and nano-micro-SiC/ZnO/EP), were prepared using nano-SiC, nano-ZnO, and micro-ZnO particles as fillers. The mass fractions of the inorganic fillers were 1, 3, and 5 wt%, respectively. The direct current (DC) voltage characteristics of the composites showed that the electrical conductivities and nonlinear coefficients of the composites utilizing single-filler types increased with increasing inorganic filler content. Under the same conditions, the conductivity and nonlinear coefficient of SiC/EP were both larger than those of the nano-ZnO/EP and micro-ZnO/EP. However, the nonlinear coefficient of the composites was significantly affected by the simultaneous addition of the two inorganic fillers, micro-ZnO and nano-SiC. When the content ratio of micro-ZnO to nano-SiC was 2:3, the nonlinear coefficient of the composite reached a maximum value of 3.506, significantly higher than those of the other samples. Compared with the nano-SiC/EP, micro-ZnO/EP and nano-ZnO/EP composites with 5 wt% inorganic filler, the nonlinear coefficient of the two-filler composite was greater by a factor of 0.82, 2.48, and 5.01, respectively.

## 1. Introduction

The non-uniform distribution of electric fields in insulating equipment and components is a widespread and difficult problem. Inhomogeneous field distribution is caused by the application of an uneven external voltage, as well as. by space charge accumulation from the hindrance of movement of free charges on the surface and in the interior of the insulating material, which results in concentrations in the local electric field [1]. In order to reduce the local electric field intensity, researchers have actively explored schemes to improve electrodes, seeking composite dielectric materials with conductivity characteristics that are affected by the applied electric field [2,3]. The conductivity of such a composite material would increase with increasing applied electric field strength, allowing alleviation of the problem of uneven electric field distribution in dielectrics [4]. Recently, researchers have focused on nanocomposites; nonlinear-conductance nanocomposites created by doping with nano-inorganic fillers have become primary topics of research and development interest [5].

Since 1990, researchers have conducted extensive studies on the effects of nanoparticle doping on polymer properties. The polymer matrices used in experimental studies have included polyethylene, polypropylene, epoxy resin (EP), and silicone rubber; nanoparticle materials have included SiO_2_, TiO_2_, ZnO, MgO, and SiC. The effects of nanoparticle doping of polymers on the composite electrical conductivity have been analyzed [6,7,8,9,10]. Researchers studying the nonlinear conductance of nano-SiC/silicone rubber composites have shown that nano-SiC doping increased the electrical conductivity of silicone rubber, and induced obvious nonlinear electrical conductivity characteristics in the composite [11,12,13]. In addition, the dielectric properties and distribution of electric field intensity of nano-SiC-modified paperboard have been simulated and analyzed. The results showed exponentially increasing conductivity in the modified insulating board with increased nano-SiC doping ratios. For high nano-SiC doping ratios, the conductivity showed strong field dependence and obvious nonlinearity [14]. Liu et al. modified an EP matrix by adding ZnO microparticles to form a new EP composite material [15]. This material also showed good nonlinear conductance characteristics. Wang et al. developed micro- and nano-Al_2_O_3_/EP composites. The experimental results showed that the nonlinear conductivity and the electrical tree growth inhibition of this material were better than those of nano-Al_2_O_3_/EP composites [16]. 

Many other scholars have studied the effects of micro- and nanoscale inorganic filler doping of dielectrics on the nonlinear conductivity characteristics [17,18,19,20,21]. However, few have investigated the effect of two inorganic fillers doped in the same dielectric. Hu et al. studied the dielectric properties of an anti-corona paint comprising a montmorillonite (MMT)/SiC micro/nanocomposite system. The results showed that with increasing SiC particle size and content, the surface resistivity of the anti-corona paint decreased, whereas the nonlinear coefficient increased [22,23]. The addition of nanoscale organic MMT effectively improved the nonlinear characteristics of the anti-corona material, reducing material loss and surface temperature under high-intensity electric fields. 

In the present paper, the nonlinear conductance properties of SiC/ZnO/EP micro/nanocomposites were investigated and compared with those of SiC/EP and ZnO/EP. The effect of two inorganic fillers doped simultaneously on the nonlinear conductivity was analyzed.

## 2. Experimental Materials and Methods

### 2.1. Raw Materials

**Figure 1:**
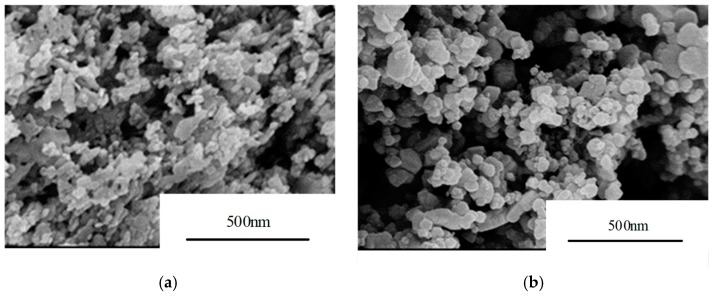
Scanning electron microscope (SEM) images of inorganic nanofillers: (**a**) nanoscale ZnO; (**b**) nanoscale SiC.

For this study, nanoscale ZnO and nanoscale SiC were purchased from Beijing DK Nano Technology Co., Ltd. (Beijing, China), with purity 99.9%, as inorganic fillers, and epoxy resin (EP) (E-44) was purchased from Nantong Xingchen Synthetic Material Co., Ltd. (Nantong, China) Scanning electron microscope (SEM, Sirion 200, FEI Technologies Inc., Hillsboro, OR, USA) images of the two inorganic fillers are shown in Figure 1.

As Figure 1a,b shows, the particle size distribution ranges of nanoscale ZnO and SiC are ~30–40 nm; thus, they are classified as nanoparticles. The nano-SiC particles are uniform in size, whereas the nano-ZnO includes small amounts of irregular particles. 

To investigate shape of inorganic particles on the effects of nonlinear conductivity characteristics of composites, microscale ZnO in needle-shapes are prepared by means of the hydrothermal method [24] in this paper. The preparation process of needle-shaped ZnO is as follows: Equal volumes of ethane diamine (C_2_H_8_N_2_) and zinc chloride (ZnCl_2_) were placed into a tri-hole bottle. The two solutions were thoroughly reacted by a magnetic stirrer and ultrasonic dispersion for 30 min. The mixed solution obtained by the reaction was added to a reaction kettle of polytetrafluoroethylene. The reaction kettle was placed in an oven at 160 °C for 12 h. After natural cooling to room temperature, a white solid powder was obtained by filtering the reaction liquid in the reaction kettle. The white solid powder was dried in a vacuum oven at 60 °C for 12 h after washing with absolute ethanol and distilled water, in this order. Finally, the micron-scale acicular ZnO was produced.

**Figure 2:**
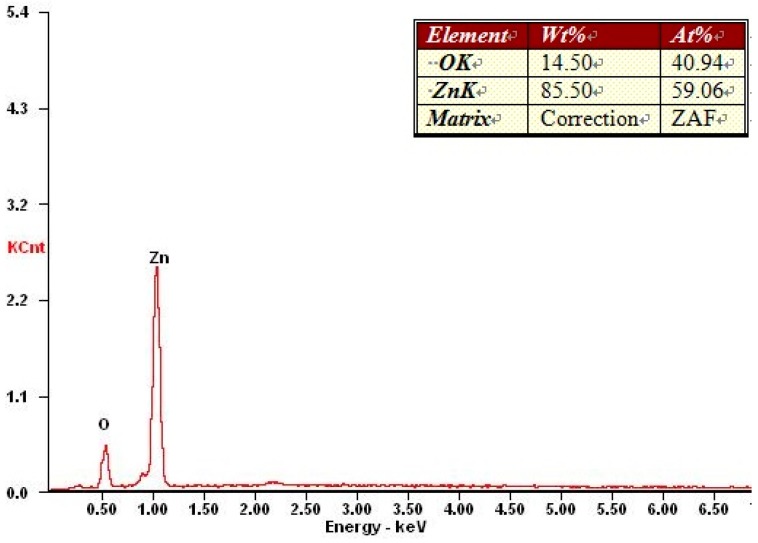
X-ray energy spectrum of ZnO.

In order to verify that the white powdery solid prepared according to the above procedure was ZnO, an X-ray energy spectrum analysis was performed; the result is shown in Figure 2. The two characteristic peaks represent the elements O and Zn. Thus it was determined that the white powdery solid prepared according to the above procedure was indeed ZnO.

**Figure 3:**
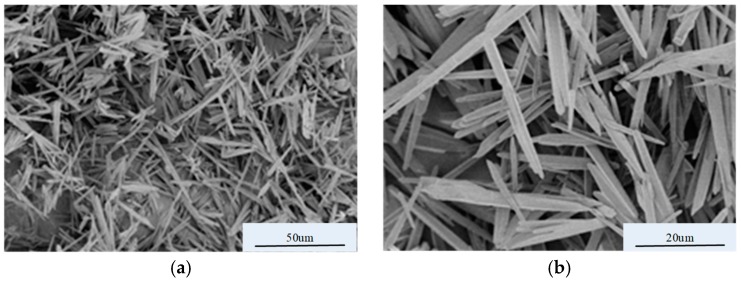
SEM images of micro-ZnO in different magnification: (**a**) magnification for scale of 50 μm; (**b**) magnification for scale of 20 μm.

The morphology of the prepared microscale acicular ZnO was observed via SEM, and the image is shown in Figure 3. As can be seen from Figure 3a, the particle shape of the prepared ZnO is acicular, the particle size is microscale, and the morphology is relatively uniform. Figure 3b provides further magnification to facilitate measurement. The micro-ZnO has particle diameters of approximately 1.5–2 μm, lengths of ~20–27 μm, and length-to-diameter ratios of ~13:1 by SEM measurement. 

### 2.2. Specimen Preparation

The micro-ZnO/EP, nano-ZnO/EP, nano-SiC/EP, nano-ZnO/nano-SiC/EP and micro-ZnO/nano- SiC/EP micro/nanocomposites were prepared by the melt blending method [25]. The particle size of the inorganic filler SiC is nanoscale. The preparation process for EP and composites was as follows:

(1) EP was preheated in a heating jacket at 80 °C for 30 min to improve its liquidity. Then, appropriate amounts of EP and the selected inorganic filler, as shown in Table 1, were placed in a three-mouth bottle, and evenly mixed by a magnetic stirrer and ultrasonic dispersion.
(2) After stirring for 3 h, an appropriate curing agent (Methylhexahydrophthalic anhydride, C_9_H_12_O_3_) and promoter (pyridine, C_5_H_5_N) were added, and magnetic stirring was continued.
(3) The above mixture was degassed, poured into a mold, and solidified in a vacuum oven at 70 °C for 1 h, and the mold was removed after natural cooling to complete the composite material preparation.

For convenience of presentation, the samples were numbered according to their inorganic filler types and mass fractions as shown in Table 1.

### 2.3. Test Method

**Figure 4:**
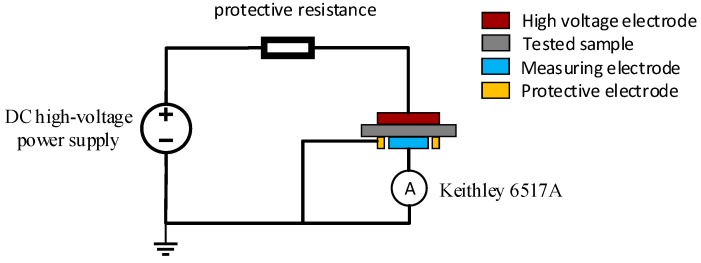
Schematic of conductance measurement system.

A three-electrode testing system was used in the experiment, with an electrode diameter of 50 mm and a protection clearance of 2 mm. Using a direct-current (DC) high-voltage power supply, the voltage was continuously adjusted from 0.3 to 15 kV. A Keithley 6517A microcurrent tester (Keithley Company, Beaverton, OR, USA) with measurement accuracy reaching 10^−15^ A was used for current testing. The specimens were round, 100 mm in diameter, 400 μm in length, and coated with an Al film. A schematic diagram of the conductance measurement system is shown in Figure 4.

According to basic dielectric physics, under an applied constant voltage, the current flowing through the dielectric gradually decreases with time before stabilizing at the conductance current. The test method usually used is to measure the conductance current flowing through the dielectric after a voltage has been applied for 1 min. Interfacial polarization and other slow polarization behaviors may exist in composites with inorganic filler under the action of DC electric fields, so the time needed for current stabilization is relatively long. During the test, the current reached a steady state in approximately 30 min, so all current values reported in this paper were measured after the voltage had been applied for 30 min.

## 3. Results and Discussion

### 3.1. Relationship between Electric Field Intensity and Conductance of Nonlinear-Conductance Materials

Conductivity is a macroscopic physical parameter, which expresses the ability of a material to conduct electricity, that is independent of dielectric shape and size, but dependent only on dielectric properties. For a dielectric sample under an applied voltage, the relationship between the current through the dielectric and the applied voltage is as shown by Equation (1):

(1) $$I=k_{1}U^{\alpha }$$

where *U* is the voltage applied on both sides of the sample (in kV), *I* is the current through the sample (in A), and *k*_1_ and α are constants.

We substitute *J* = *I*/*S* (where *S* is the effective area of the measuring electrode in mm^2^), *γ* = *J*/*E* (where *E* is the electric field intensity in kV/mm), and *E* = *U*/*d* (where *d* is the thickness of the specimen in mm) into Equation (1) to obtain

(2) $$\gamma =kE^{\alpha -1}$$

where *γ* is the conductivity (in S/m).

In order to study the nonlinear conductivity of composite materials, the nonlinear coefficient *β* (*β* = *α* − 1) is defined to describe the dependence of the conductivity on the electric field intensity. The logarithm of both sides of Equation (2) is taken to yield

(3) lgγ=lgk+βlgE

where *β* is the nonlinear coefficient of conductance.

Equation (3) shows that in double-logarithmic coordinates, the conductivity *γ* of the nonlinear material and the electric field intensity *E* have a linear relationship. Therefore, after obtaining the test data *I* and *U*, the above relation can be used to process the data to obtain the nonlinear conductivity coefficient *β.*

Nonlinear-conductance materials have critical electric field intensities, also called threshold field intensities. For an applied electric field intensity below the threshold field intensity, the change in conductance and the nonlinear coefficient are very small. 

With an externally applied electric field of an intensity greater than the threshold field intensity, there is an obvious change in conductance with the change in electric field intensity; thus, the conductance shows nonlinear characteristics. 

Because the EP matrix conductivity arises from the interaction of multiple factors, the introduction of inorganic micro/nanoparticles to the composite system produces interfacial effects according to the multi-core model theory [26,27]. The interface areas overlap, forming conductive channels, which cause changes in the conductivity characteristics of the composite. 

### 3.2. Effect of Inorganic Filler Concentration on the Conductivity of EP Composites

**Figure 5:**
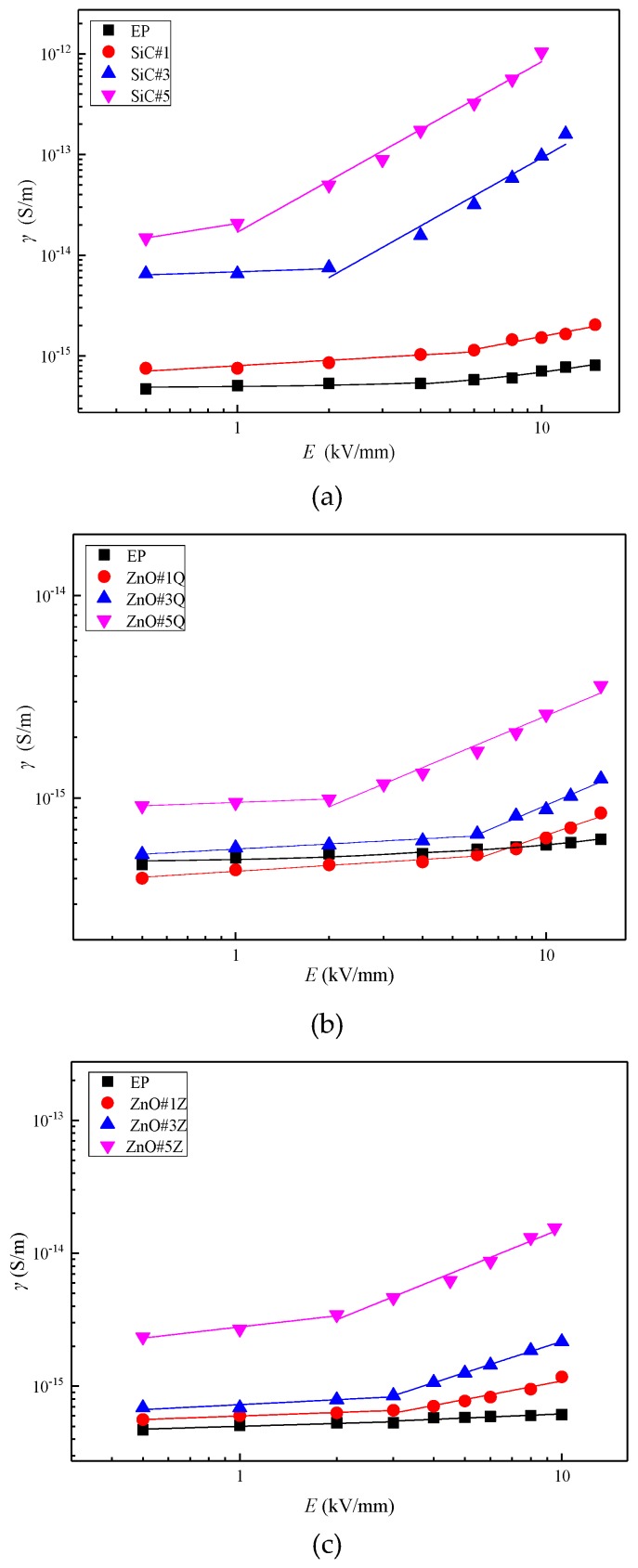
*γ*–*E* characteristic curves of composites for different filler concentrations (with respect to epoxy resin (EP)): (**a**) nano-SiC/EP composites; (**b**) nano-ZnO/EP composites; (**c**) micro-ZnO/EP composites.

The *γ*–*E* characteristic curves of the three types of single-filler samples are shown in Figure 5. 

Figure 5 shows that the variations of the *γ*–*E* characteristic curves of the three types of composite materials are similar. The threshold field intensity decreases with an increasing inorganic filler mass fraction, whereas the conductivity increases with an increasing filler mass fraction. For the 1 wt% nano-ZnO/EP composite, the sample conductivity is lower than that of the pure EP material. This is mainly because interface structures are formed with ZnO nanoparticles at the center; these capture some charge carriers, causing decreased sample conductivity at low electric field intensity.

**Figure 6:**
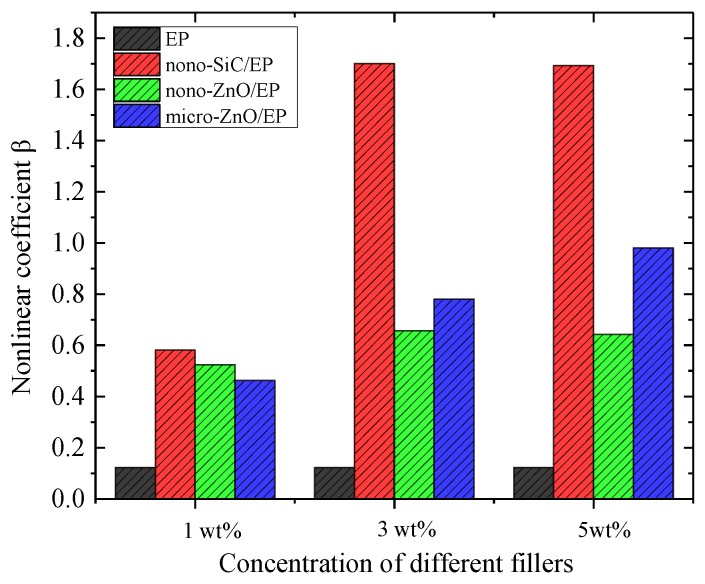
Nonlinear coefficients of three composite materials at different mass fractions.

For externally applied electric fields with intensities higher than the threshold field intensity, the sample nonlinear coefficients are as shown in Figure 6.

The nonlinear coefficients of inorganic-filler-doped EP are significantly higher than that of undoped EP, and the nonlinear coefficients increase with increasing dopant concentrations. At equal doping concentrations, the nonlinear coefficients of the nano-SiC/EP composites are the highest. At 5 wt% inorganic filler, the nonlinear coefficient of the SiC/EP composite is 1.69, which is 1.72 times that of the micro-ZnO/EP composite and 2.64 times that of the nano-ZnO/EP composite.

Without inorganic filler, fewer carriers are present in the internal medium of the EP. Under a high electric field, electrons are easily injected into the sample by the electrode or ionized inside the material to generate electronic conductance. Therefore, the conductivity of undoped EP also shows nonlinearity; however, its threshold field intensity is large and the nonlinear coefficient is very small for externally applied electric fields exceeding the threshold field intensity. After the addition of inorganic filler, the nanoparticles agglomerate to form groups distributed in the EP matrix. According to percolation theory [28], at a certain concentration of inorganic filler, conductive groups form in the matrix. Because the loose layers of the interfacial structures overlap, conductive channels form easily and thus the carrier mobility, i.e., the conductivity, increases. At this point, the nonlinear coefficient increases obviously due to the change of conduction mechanism. The nonlinearity of a composite with filler is mainly due to the effective contact interface between filler particles dispersed in the polymer matrix. The space charges accumulating at the interface between the filler particles should cause bending of energy bands in the composites, which leads to forming of Schottky barriers in back-to-back type at the particle contacts. It make the composite exhibit better nonlinear conductivity characteristics, as seen in Figure 6. However the nonlinear coefficients of SiC/EP and ZnO/EP nano-composites change litter when the content of inorganic fillers increase from 3 wt% to 5 wt%, which may be due to a percolation phenomenon. When the interfaces between the filler particles have formed an effective conduct path in the composites, more filler particles are not necessary, and it is even possible that the nonlinear coefficient reduces as the conductivity increases [5].

Carrier migration between different conductive groups requires a certain electric field strength. With uniformly dispersed conductive groups, carriers can migrate between most groups with electric fields reaching the threshold field intensity; this causes the electrical conductivity of the composite to increase rapidly. 

The nonlinear coefficient of the composite material changes significantly near the threshold field intensity. The distance between conductive groups decreases with increasing inorganic filler concentration, allowing carrier migration at lower electric field strengths. Therefore, the threshold field strength decreases, and the conductivity increases as the filler concentration increases. 

### 3.3. Effect of Inorganic Filler Type on the Conductivity of EP Composites

**Figure 7:**
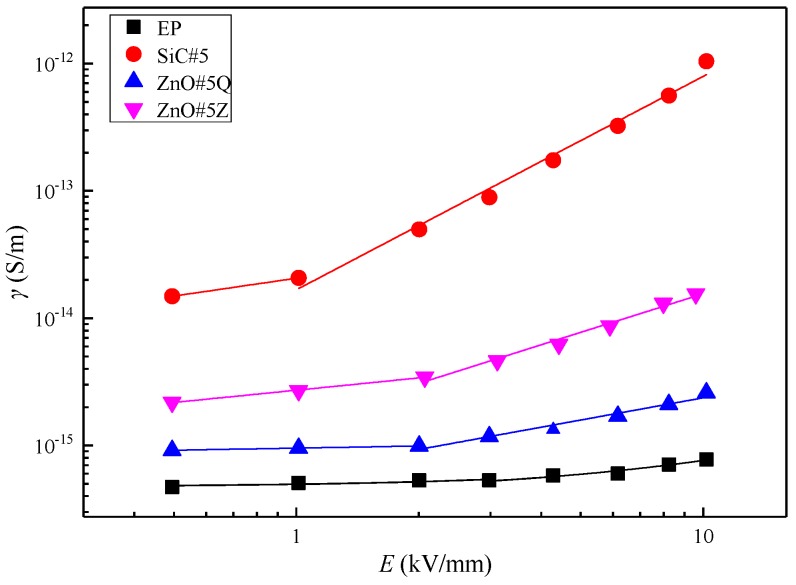
*γ*–*E* characteristic curves of composites with 5 wt% inorganic filler.

According to the above experiments, at 5 wt% inorganic filler, the *γ*–*E* characteristic curves of the composite materials are as shown in Figure 7. 

**Figure 8:**
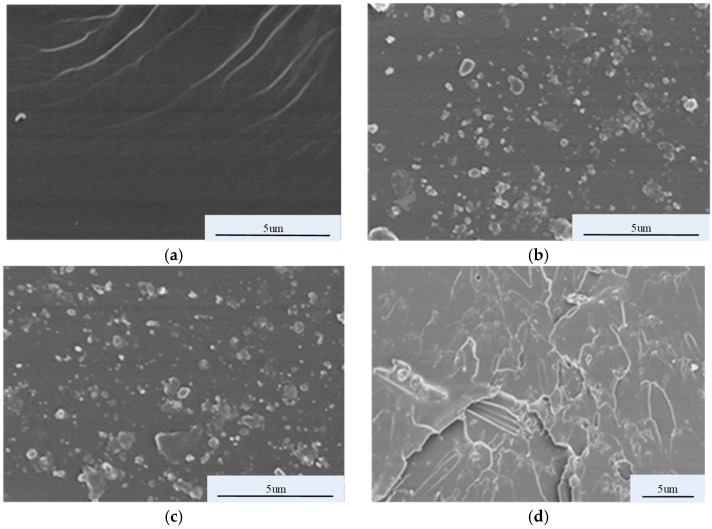
SEM images of undoped epoxy resin (EP) and EP doped with individual inorganic filler types: (**a**) undoped EP; (**b**) SiC#5; (**c**) ZnO#5Q; (**d**) ZnO#5Z.

Figure 7 shows that at 5 wt% inorganic filler, the electrical conductivities of the composite samples decrease in the order of SiC/EP, micro-ZnO/EP, and nano-ZnO/EP. The conductivities of the three composite materials are higher than that of pure EP. SEM images of the four materials in Figure 7 are shown in Figure 8.

In the SEM images, the dark areas are the EP matrices and the bright parts are the inorganic filler. No bright regions are observed in Figure 8a, which depicts the pure EP. Figure 8b–d indicates that each inorganic filler type is well dispersed in the EP matrix without obvious agglomeration and with good EP combination. A comparison of Figure 8c,d shows that micro-ZnO forms conductive networks across the sample more easily than nano-ZnO does, thus facilitating carrier migration. Therefore, the conductivity of micro-ZnO/EP composites is greater than that of nano-ZnO/EP composites. 

### 3.4. Effect of the Ratio of SiC and ZnO Inorganic Fillers on the Conductivity of EP Composites

**Figure 9:**
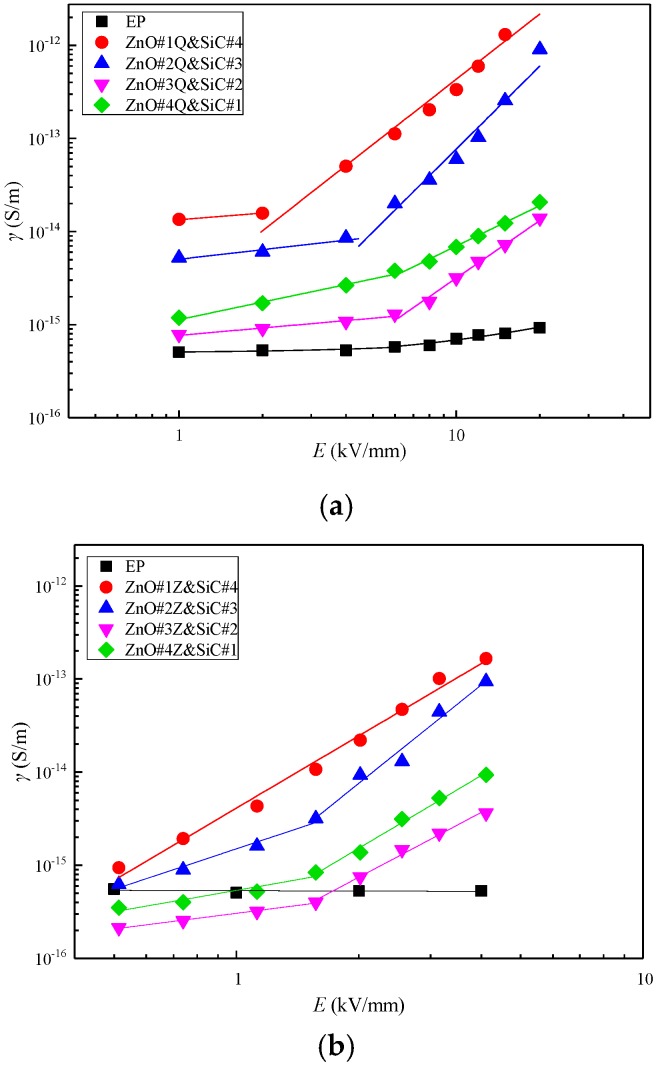
*γ*–*E* characteristic curves of composite materials with different proportions of inorganic fillers: (**a**) nano-ZnO/SiC/EP nanocomposites; (**b**) micro-ZnO/SiC/EP micro/nanocomposites.

**Figure 10:**
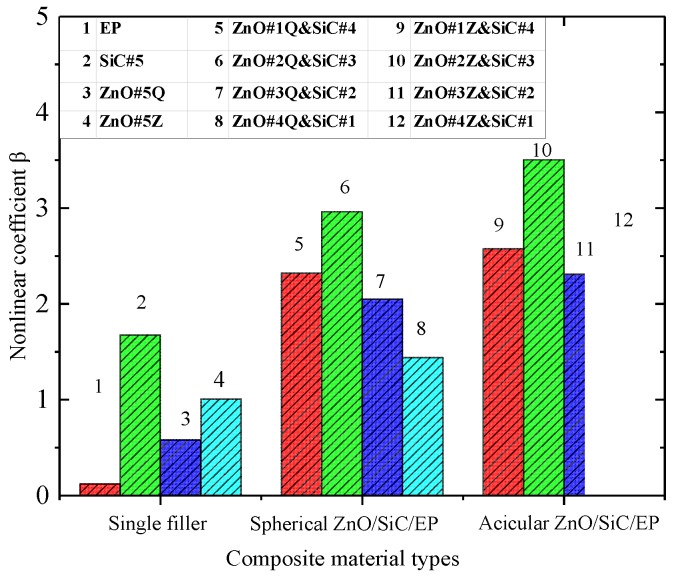
Nonlinear coefficients of composites with different inorganic fillers.

In order to explore the influence of the addition of inorganic micro- and nanoparticle filler on the conductivity characteristics of the composites, micro-ZnO/SiC/EP micro/nanocomposite and nano-ZnO/SiC/EP nanocomposite were prepared. In both types, the total mass fraction of inorganic filler was 5 wt%. The ratios of ZnO to SiC were 1:4, 2:3, 3:2, and 4:1. The characteristic curves of *γ*–*E* are shown in Figure 9, and the nonlinear coefficients of conductivity are shown in Figure 10. 

It can be seen from Figure 9 that both composite materials show increased electrical conductivity and decreased threshold field strength with increasing SiC concentration. Therefore, SiC is considered dominant in determining the conductance characteristics of the ZnO/SiC/EP composites. Figure 10 shows that the nonlinear coefficients of conductance of the materials using two inorganic filler types are obviously superior to those of single-filler composites. At the ZnO: SiC ratio of 2:3, the nonlinear coefficients of nano-ZnO/SiC/EP and micro-ZnO/SiC/EP reach maximum values of 2.963 and 3.506, respectively. Therefore, the micro-ZnO inorganic filler has a greater influence on the nonlinear coefficient. Compared with the composite materials of 5 wt% SiC/EP, micro-ZnO/EP, and nano-ZnO/EP, the nonlinear coefficient of the 5 wt% 3:2 micro-ZnO/SiC/EP composite is greater by a factor of 0.82, 2.48, and 5.01, respectively.

**Figure 11:**
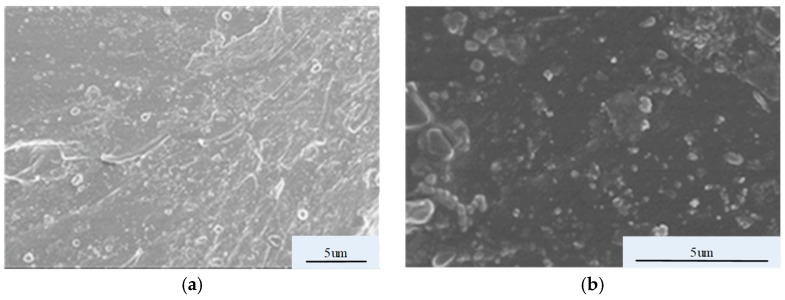
SEM images of EP doped with two kinds of inorganic filler: (**a**) micro-ZnO#2Z&SiC#3; (**b**) nano-ZnO#2Q&SiC#3.

SEM images of the composite materials with a 2:3 ratio of ZnO to SiC are shown in Figure 11.

As seen in Figure 11a, in the micro-ZnO/SiC/EP micro/nanocomposite, the two inorganic fillers have good dispersion with nano-SiC evenly distributed around the micro-ZnO. Micro-ZnO has long, microscale particles. In the EP matrix with nano-SiC, it forms an interfacial structure within the matrix that captures some carriers under low field strengths. This decreases the mobility; therefore, the conductivity is slightly lower than that of pure EP. With an increase in electric field intensity, the conductive channels in the interfacial structure become more important and the carrier mobility is increased. Because of the length of the microscale acicular ZnO particles, conductive channels are easily formed in the EP matrix for carrier migration, which significantly decreases the threshold field intensity and rapidly increases the conductivity. This is also why micro-ZnO has a large impact on the nonlinear coefficient. Nano-SiC has good electrical conductivity, and so the combination of SiC and micro-ZnO forms good conductive pathways in the EP body, facilitating carrier migration between different conductive groups. As seen in Figure 11b, agglomeration is more likely between the smaller-sized nano-ZnO and SiC particles, the spherical particles are separated by a certain distance, and few effective conductive channels are formed. This yields a conductivity in the nano-ZnO/SiC/EP nanocomposites lower than that in the micro-ZnO/SiC/EP micro/nanocomposites under high electric fields.

## 4. Conclusions

From our analysis of the results of our study, we offer the following conclusions:

(1) At the same electric field intensity, the conductivity of SiC/EP composites is greater than that of micro-ZnO/EP composites, which is greater than that of nano-ZnO/EP composites.
(2) The composite conductivity increases with increased inorganic filler content. At equal content levels, the nonlinear coefficients of SiC/EP composites are greater than those of micro-ZnO/EP and nano-ZnO/EP composites.
(3) When the ratio of micro-ZnO to SiC is 2:3, the nonlinear coefficient of the composite material above the threshold field intensity is maximized at 3.506, which is significantly larger than that of any single-filler sample. Compared with the SiC/EP, micro-ZnO/EP, and nano-ZnO/EP composite materials with equal filler content levels, the nonlinear coefficient is greater by a factor of 0.82, 2.48, and 5.01, respectively. The threshold field intensity of the micro-ZnO and SiC micro/nanocomposite material is clearly reduced.

## Figures and Tables

**Table 1:** Sample names and main components.

Sequence Number	Simplified Name of Sample	Nano-ZnO Mass Fraction (wt%)	Micro-ZnO Mass Fraction (wt%)	Nano-SiC Mass Fraction (wt%)	Sample Category
1	EP	-	-	-	EP
2	ZnO#1Q	1	-	-	ZnO/EP nanocomposite
3	ZnO#3Q	3	-	-	
4	ZnO#5Q	5	-	-	
5	ZnO#1Z	-	1	-	ZnO/EP microcomposite
6	ZnO#3Z	-	3	-	
7	ZnO#5Z	-	5	-	
8	SiC#1	-	-	1	SiC/EP nanocomposite
9	SiC#3	-	-	3	
10	SiC#5	-	-	5	
11	ZnO#1Q&SiC#4	1	-	4	SiC/ZnO/EP nanocomposite
12	ZnO#2Q&SiC#3	2	-	3	
13	ZnO#3Q&SiC#2	3	-	2	
14	ZnO#4Q&SiC#1	4	-	1	
15	ZnO#1Z&SiC#4	-	1	4	SiC/ZnO/EP micro/nanocomposite
16	ZnO#2Z&SiC#3	-	2	3	
17	ZnO#3Z&SiC#2	-	3	2	
18	ZnO#4Z&SiC#1	-	4	1	
